# Semaphorin 3A Shifts Adipose Mesenchymal Stem Cells towards Osteogenic Phenotype and Promotes Bone Regeneration In Vivo

**DOI:** 10.1155/2016/2545214

**Published:** 2016-09-19

**Authors:** Xiangwei Liu, Naiwen Tan, Yuchao Zhou, Xueying Zhou, Hui Chen, Hongbo Wei, Ji Chen, Xiaoru Xu, Sijia Zhang, Guodong Yang, Yingliang Song

**Affiliations:** ^1^State Key Laboratory of Military Stomatology and National Clinical Research Center for Oral Diseases and Shaanxi Engineering Research Center for Dental Materials and Advanced Manufacture, Department of Implant Dentistry, School of Stomatology, Fourth Military Medical University, Xi'an 710032, China; ^2^Department of Ultrasound Diagnostics, Tangdu Hospital, Fourth Military Medical University, Xi'an, China; ^3^Department of Plastic Surgery, Tangdu Hospital, Fourth Military Medical University, Xi'an, China; ^4^Department of Biochemistry and Molecular Biology, Fourth Military Medical University, Xi'an, China

## Abstract

Adipose mesenchymal stem cells (ASCs) are considered as the promising seed cells for bone regeneration. However, the lower osteogenic differentiation capacity limits its therapeutic efficacy. Identification of the key molecules governing the differences between ASCs and BMSCs would shed light on manipulation of ASCs towards osteogenic phenotype. In this study, we screened semaphorin family members in ASCs and BMSCs and identified Sema3A as an osteogenic semaphorin that was significantly and predominantly expressed in BMSCs. The analyses in vitro showed that the overexpression of Sema3A in ASCs significantly enhanced the expression of bone-related genes and extracellular matrix calcium deposition, while decreasing the expression of adipose-related genes and thus lipid droplet formation, resembling a BMSCs phenotype. Furthermore, Sema3A modified ASCs were then engrafted into poly(lactic-co-glycolic acid) (PLGA) scaffolds to repair the critical-sized calvarial defects in rat model. As expected, Sema3A modified ASCs encapsulation significantly promoted new bone formation with higher bone volume fraction and bone mineral density. Additionally, Sema3A was found to simultaneously increase multiple Wnt related genes and thus activating Wnt pathway. Taken together, our study here identifies Sema3A as a critical gene for osteogenic phenotype and reveals that Sema3A-modified ASCs would serve as a promising candidate for bettering bone defect repair.

## 1. Introduction

Bone defects are common in trauma, tumor resection, or congenital local bone absence, among which approximately 5–10% could not heal spontaneously due to excessive bone loss or abnormal bone metabolism [1]. Autogenous bone graft or bone graft substitutes are needed in this situation [2, 3]; however, sometimes autografting is insufficient or problematic in certain conditions of great bone trauma or systemic diseases [2, 3]. Therefore, bone graft substitute is a promising alternative [2, 3]; in particular, tissue engineering derived bone consisting of seed cells, scaffolds, and growth factors is becoming a new trend in the field. As to the seed cells, bone marrow mesenchymal stem cells (BMSCs) are the first rational seed cells for bone defect repair and regeneration, while the sources of BMSCs are limited [4, 5]. In 2002, Zuk et al. [6] found a new source of seed cells, adipose-derived stem cells (ASCs), which are readily available, able to differentiate into osteocytes, and potent in replication [7–9]. Compared with the BMSCs, ASCs have a poor performance in osteogenesis due to their origin, with the detailed mechanism unknown [10–13]. Approaches to increase the osteogenic capacity of ASCs would possibly facilitate the utility of ASCs in bone repair and regeneration.

Previously, a number of approaches aiming at increasing the osteogenic capacity of ASCs were tried. ASCs are heterogeneous cells populations, with the osteogenic and self-renewal capacities being different [14–16]. To this end, the ASCs subpopulations with strong osteogenesis potential were sorted via flow cytometry [17] and utilized for bone engineering. Besides, osteogenic stimuli are added to promote or activate the bone forming capacity of ASCs before transplantation. For example, short-time exposure to osteogenic medium of ASCs would upgrade the role in bone defect healing, and this pretreatment would also prevent tumor formation theoretically [18]. Besides, many other biological factors, such as osteoprotegerin, platelet-derived growth factor BB and bone morphogenetic protein, loaded directly or on various vehicles for control release, have also been tried, with different beneficial outcomes [19, 20]. Clarification of the mechanisms underlying the osteogenic differences between BMSC and ASCs would provide clues for improving the osteogenesis of ASCs, shedding light on the bone regeneration.

Semaphorin family, composed of more than twenty secreted and membrane-associated proteins, plays a critical role in development and wound repair, such as neural axonal guidance, angiogenesis, and bone formation [21]. Accumulating studies indicate that some semaphorin family members are involved in bone remodeling by promoting osteogenesis [22–28], further suggesting the potent role of semaphorin in bone development and regeneration. Up to now, whether semaphorin is responsible for the difference between ASC and BMSC in osteogenesis is totally unknown.

In this study, we compared the expression of semaphorin members in mesenchymal stem cells with varied osteogenic capacities, namely, young BMSCs, aged BMSCs, and ASCs. Semaphorin 3A was identified as a gene highly expressed in the mesenchymal stem cells with strong osteogenic capacity. In vitro studies further confirmed the osteogenic role of Sema3A by both Sema3A knockdown and overexpression. Transplantation of Sema3A infected ASCs greatly improves the bone repair and regeneration capacities in the calvarial critical size bone defect model. Our study has revealed that manipulating ASCs with Sema3A holds as a promising approach for bone repair and regeneration in the future.

## 2. Materials and Methods

### 2.1. Cell Isolation and Culture

Sprague Dawley (SD) rats (4 weeks or 12 months old), obtained from the animal center of Fourth Military Medical University (FMMU), were grown and processed in accordance with Institutional Animal Care and Use Committee (IACUC) approval of FMMU. For ASCs isolation, the rats were sacrificed by euthanization followed by cervical dissection. The bilateral inguinal adipose tissues were harvested under sterile conditions and then digested with 0.2% collagenase I for 40 mins and filtered with 100 *μ*m strainer mesh. The obtained filtrate was centrifuged (5 min, 1000 r/min) and resuspended in DMEM medium (GIBCO, USA) containing 10% fetal bovine serum (GIBCO, USA) and 1% antibiotics (200 mg/mL penicillin and 200 mg/mL streptomycin,) and cultured in an humidified atmosphere of 5% CO_2_ at 37°C. ASCs were passaged upon reaching 80% confluency. For BMSCs isolation, bone marrows of the femur and tibia were harvested by syringe flushing, and then the pelleted cells were resuspended and cultured in the same way above. Passage 3 cultures were applied for both in vivo and in vitro experiments in this study.

### 2.2. Lentivirus Packaging and Infection of ASCs

The lentivirus plasmid expressing Sema3A gene was constructed by cloning the Sema3A CDS into pWPI vector, designated as pWPI-Sema3A. Lentivirus expressing Sema3A was packaged by transfecting 293T cells with pWPI (or pWPI-Sema3A), psPAX2, and pMD2G plasmids at the mass ratio of 10 : 5 : 1. The obtained lentivirus expresses Sema3A and green fluorescent protein (GFP) in pWPI-Sema3A or in control pWPI. For lentivirus infection, the obtained lentivirus was incubated with polybrene (8 *μ*g/mL) before being added into the ASCs. To define the MOI, ASCs were seeded at a density of 4 × 10^5^/well into a six-well plate in average DMEM medium and infection efficiency was determined by flow cytometric detection of GFP expression.

### 2.3. Flow Cytometric Analysis

The infection efficiency was analyzed by flow cytometry. Forty-eight hours after infection, approximately 3 × 10^5^ ASCs either without infection or infected with control pWPI lentivirus or Sema3A expressing lentivirus were digested with trypsin/EDTA and resuspended in 500 *μ*L PBS. The expression of GFP in the above three groups was analyzed by flow cytometry (BD, FACSAria).

### 2.4. Western Blot

Whole-cell protein was isolated from ASCs using the Total Extraction Sample Kit (Sigma) according to the instruction. For western blot analysis, equal proteins were loaded on 12% SDS-PAGE gels and then separated completely using vertical electrophoresis at 120 V. After transferring onto a 0.5 *μ*m pore size nitrocellulose membrane at 200 mA, the separated proteins were blocked with 5% bovine serum albumin and incubated with rabbit polyclonal anti-Sema3A(Abcam, ab23393) and mouse polyclonal anti-GAPDH primary antibodies diluted in 5% TBST for 8 hours at 4°C. The protein bands were visualized using the Odyssey Infrared Imager system (LI-COR) after incubation with corresponding fluorescent secondary antibody for 1 hour at room temperature.

### 2.5. Cell Proliferation and Migration Assay

For cell proliferation analysis, two thousand cells with indicated treatments in 150 *μ*L medium were seeded per well in 96-well plate. Ten *μ*L cck-8 reagents were gently added and mixed evenly into each well at indicated time. After 2 hours of incubation at 37°C, the absorbance value was read at 490 nm. For cell migration assay, about 20,000 mesenchymal stem cells were seeded on the Transwell insert with 8 *μ*m pore size followed by surface wetting utilizing serum-free medium for 1 hours, with the bottom chamber containing 500 *μ*L Sema3A ASC conditioned or control medium. Forty-eight hours later, migrated cells were fixed and stained with crystal violet. The migrated cells were visualized and counted under inverted microscope.

### 2.6. Adipogenic and Osteogenic Differentiation Induction

ASCs were maintained in the growth medium till confluence. One day after confluence, ASCs were converted into adipogenic induction medium (50 *μ*M 3-isobutyl-1-methylxanthine (Sigma-Aldrich), 10 *μ*M dexamethasone (Sigma-Aldrich), 10 *μ*M rosiglitazone (Sigma-Aldrich), and 10 *μ*g/mL insulin). The medium was changed every 3 days. The differentiated cells were fixed in 4% formaldehyde for 15 minutes at room temperature and lipid droplets were identified by Oil red O (Sigma-Aldrich) staining. For osteogenic differentiation, 70% confluent cells were induced by osteogenic induction medium containing 10 mM disodium *β*-glycerophosphate (Sigma-Aldrich), 0.1 *μ*M dexamethasone (Sigma-Aldrich), and 50 mg/mL l-ascorbic acid (Sigma-Aldrich). The medium was also changed every 3 days and full osteogenic differentiation was achieved by 3-week induction, during which time we measured alkaline phosphatase (ALP) activity of ASCs and performed ALP staining after a 7-day osteogenic differentiation induction, using ALP assay kit and ALP color development kit. Osteogenic differentiation was examined by Alizarin Red (Sigma-Aldrich) staining of mineralized matrix. The CPC (cetylpyridinium chloride, Sigma-Aldrich) method was used to quantify the mineralized matrix as described elsewhere [29]. Briefly, after staining with Alizarin Red, CPC (10%, w/v, in distilled water) solution was added to each sample (800 *μ*L per well in 24-well plate) and incubated for 1 h at 37°C. After incubation, the CPC solution was transferred to a 96-well plate (80 *μ*L per well) for absorbance reading at 570 nm.

### 2.7. Real-Time Quantitative PCR Analysis

Total RNA was isolated using the Trizol (Invitrogen) before being subjected to reverse transcription using PrimeScript RT Reagent kit (Takara). Real-time PCR analysis (qPCR) was conducted with SYBR Premix Ex Taq II (Takara) using a CFX96 Real-Time System (Bio-Rad). GAPDH or *β*-actin was selected as the internal control. The referred primer sequences are listed in Supplementary  Table 1 in Supplementary Material available online at http://dx.doi.org/10.1155/2016/2545214.

### 2.8. Topflash Luciferase Reporter Assay

Wnt activity was analyzed by Topflash analysis. Briefly, ASCs^-control^ and ASCs^-Sema3A^ were, respectively, plated in 24-well plates at a density of 1 × 10^5^ cells per well and cultured for 24 hours. Then, the 200 ng Topflash or Fopflash plasmid together with 10 ng pGL4.75 plasmid was transfected using Lipofectamine 2000 Transfection Reagent. Forty-eight hours after transfection, the cells were lysed and the relative luciferase activities were measured using Dual Luciferase Reporter Assay System according to the protocol.

### 2.9. Immunofluorescence

Immunofluorescence was included for probing the subcellular location of *β*-catenin in ASC^-Sema3A^ and control ASCs. Briefly, the cells were fixed for 15 minutes in 4% formaldehyde and blocked with PBS containing 3% BSA and 0.3% tween for 1 h. After that, cells were incubated with primary anti-*β*-catenin antibody (ab32572, 1 : 200, Abcam) overnight at 4°C. The secondary antibody Alexa Fluor 555 goat anti-rabbit IgG antibody was added for 1 hour to visualize primary antibody at room temperature. Nuclei were counterstained in blue with Hoechst (1/1000). Images were visualized with confocal microscope (Nikon, Japan).

### 2.10. Construction of PLGA Scaffold-ASCs Implant

Poly(lactic-co-glycolic acid) copolymer-collagen scaffolds were fabricated as described before [16], with minor modification. Briefly, ten-gram NaCl particles varying from 125 *μ*m to 300 *μ*m in diameter were seeded evenly in an 8 cm glass dish. And one gram 65 : 35 PLGA (Sigma-Aldrich) was dissolved entirely in 9 mL dichloromethane (DCM). Then, the PLGA solution was poured into the glass dish, and another ten-gram NaCl particles were added into the glass as soon as the liquid level sank down to NaCl particles. All the procedure was done in the chemical hood and the DCM was removed by evaporation. NaCl was removed by flushing with large amounts of water. Five mm diameter PLGA discs were obtained utilizing electric trephine. The obtained discs were sterilized by Co60 irradiation and further immersed into 50 mg/mL collagen I solution for one hour to increase the compatibility with cells. The ASCs-scaffold complex was manipulated by dropping 50 *μ*L ASCs solution (2 million cells) onto the scaffold. The surface morphology of empty scaffold and ASCs encapsulated scaffold were analyzed by scanning electron microscopy. The acquired implants were cocultured in the osteogenic medium for 48 hours before being transplanted in vivo.

### 2.11. Creation of the Critical Size Calvarial Defect Model and Transplantation of ASCs-Scaffold Implant

Adult male SD rats, weighing 250–300 g, were intraperitoneally injected with pentobarbital (1%, 0.4 mL/100 g) and then subjected to local anesthesia with primacaine. The surgical field was shaved and sterilized; 2 cm long incisions were made approximately from the occipital middle region to the nose. Using a trephine with saline irrigation cooling, 5 mm full-thickness bone defects were created. The rats were randomly divided into four groups according to the differences of implants: (1) control group, (2) PLGA scaffold only, (3) PLGA scaffold with ASCs^-control^, and (4) PLGA scaffold with ASCs^-Sema3A^. Following suture and postoperative anti-inflammation treatment with gentamycin, animals were kept for 4- and 16-week recovery before microCT and histology study.

### 2.12. Histology and Immunohistochemistry

To analyze the cell survival and cell migration on the scaffolds, PLGA scaffold with different fabrications was taken out from the defects five days after implantation. Then, the complexes were embedded in paraffin and cut into 5 *μ*m sections. Immunohistochemistry was carried out as reported by Zavatti et al. and so forth [30]. Briefly, the sections were treated with boiling EDTA buffer to expose the antigens. Then the sections blocked with 3% BSA were further incubated with the rabbit anti-collagen I antibody for 1 hour at RT. After 3 times washing, the samples were incubated for 1 hour at RT with the secondary antibody diluted 1 : 200 before DAB staining. After washing again, the sections were counter stained with hematoxylin.

For histological analysis after 4- or 12-week recovery, the decalcified specimens were embedded in paraffin and then cut into 5 *μ*m sections. The sections were stained with hematoxylin and eosin (H&E) to highlight the different tissues. The digital sections images were captured with the aid of light microscope.

### 2.13. Micro-CT Scanning

After being intraperitoneally injected with excessive pentobarbital, rats were sacrificed and the calvariae around the bone defect were excised and placed in 10% neutral buffered formaldehyde for 12 hours. For further imaging examination, a high-resolution micro-CT scanner was employed. Samples were scanned with a resolution of 20 *μ*m; afterwards, whole cranium was reconstructed using data analysis software. Five mm in diameter along the edge of defect was set as the region of interest. The bone volume fraction (BVF) and the trabecular thickness were calculated.

### 2.14. Statistical Analysis

All data were expressed as mean ± SD. Statistical significance was determined with Student's* t*-test or ANOVA using SPSS software. Statistical significance was accepted for *p* < 0.05.

## 3. Results

### 3.1. Specific High Expression of Sema3A in Young BMSCs Correlates with the Potent Osteogenic Capacity

**Figure 1:**
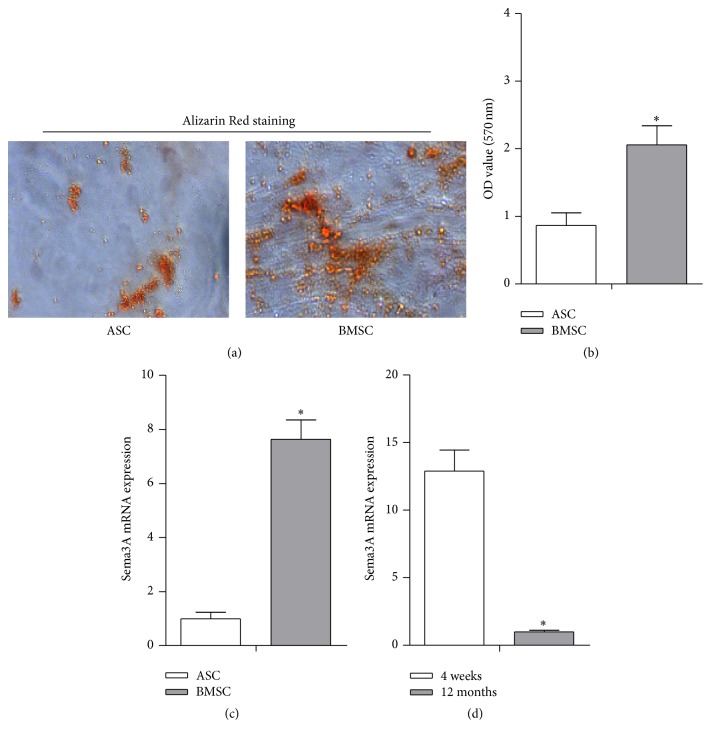
High expression of Sema3A correlates with the potent osteogenic capacity in BMSCs. (a) ASCs and BMSCs from the same rat were cultured in the osteogenic medium and significantly less mineralization nodules were found in ASCs than that in BMSCs after 21 days of osteogenic induction. (b) Quantification data of ECM mineralization in Figure 1(a). (c) Sema3A expression was significantly less in ASCs than that in BMSCs, as detected by qPCR. (d) Lower Sema3A expression was also found in 12-month rat derived BMSCs, compared with that from 4-week rat derived BMSCs. Mean ± SD, *n* = 3, and ^*∗*^ *p* < 0.05. Sema3A, semaphorin 3A; ECM, extracellular matrix.

Consistent with previous findings that BMSCs have stronger osteogenic capacity than ASCs, Alizarin Red staining confirmed that the BMSCs were more likely differentiated into osteoblasts (Figures 1(a) and 1(b)). To reveal the molecular determinants for the phenotype differences, we mainly focus on the semaphorin family, which has been reported to play important roles in cell fate commitment [31, 32]. We screened the expression of semaphorin family members (semaphorins 3A, 3B, 3C, 3D, 3E, 3F, 4A, 4B, 4C, 4D, 4F, 4G, 5A, 5B, 6A, 6B, 6C, 6D, and 7A) in ASCs and BMSCs and found strikingly higher expression of Sema3A and Sema3D in BMSCs (Figures 1(c) and 1(d), Supplementary Figures  1A-B). Sema3A decreased with aging, while Sema3D did not change significantly in the aging process (Figures 1(c) and 1(d), Supplementary Figures  1A-B). In addition, the absolute abundance of Sema3D in BMSCs was much smaller than Sema3A in BMSCs (data not shown). Taken together, we proposed that Sema3A might be an important molecular determinant for the osteogenic differences between ASCs and BMSCs.

### 3.2. Overexpression of Sema3A Shifts the Cells towards Osteogenic Phenotype

In view of the above-mentioned data, we next explored the possible role of Sema3A in osteogenesis and adipogenesis. Control and Sema3A expressing lentivirus were packaged using the pWPI lentivirus system. ASCs were infected with the lentivirus at MOI = 100, and the infection efficiency was confirmed by the simultaneous expression of EGFP as detected by flow cytometry (Supplementary Figure  2A). Q-PCR and western blot analysis further confirmed the overexpression efficiency (Supplementary Figures  2B-C).

**Figure 2:**
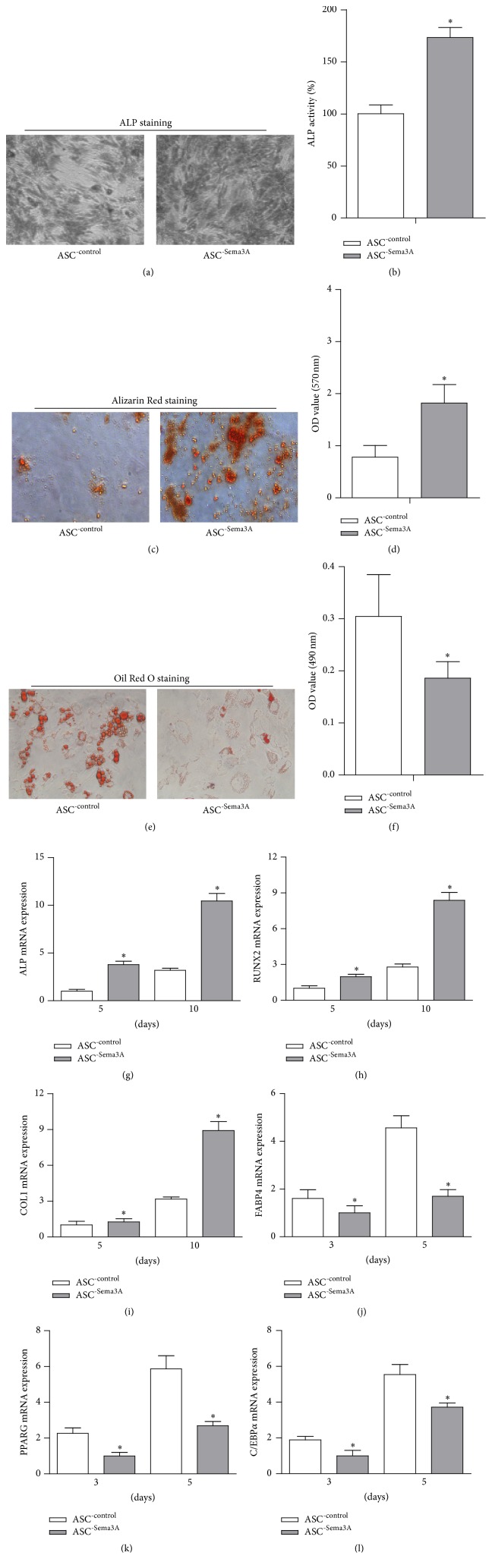
Sema3A promotes osteogenic differentiation while inhibiting adipogenic differentiation in ASCs. (a) ASCs were infected with either empty pWPI or Sema3A overexpression lentivirus, respectively. ASC^-Sema3A^ showed higher ALP activity than ASC^-control^ after 7-day osteogenic induction. (b) Quantification data of ALP activity. (c) Significantly more mineralization nodules were found in ASC^-Sema3A^ group than that in ASC^-control^ group after 21 days of osteogenic induction. (d) Quantification data of ECM mineralization in Figure 2(c). (e) Sema3A significantly reduced lipid droplet formation in ASCs after 9 days of adipogenic induction. (f) Quantification data of lipid droplets in Figure 2(e). (g, h, and i) Expression of the osteogenesis related genes including ALP, RUNX2, and COL-1 after 5 and 10 days of osteogenic induction. (j, k, and l) Expression of the adipogenesis related genes including FABP4, PPARG, and C/EBPA after 3 and 5 days of adipogenic induction. Mean ± SD, *n* = 3, and ^*∗*^ *p* < 0.05.

With the increase of Sema3A in ASCs, both ALP activity and deposition of calcified extracellular matrix were increased, as detected by ALP assay and Alizarin Red staining results (Figures 2(a)-2(d)). In contrast, Oil Red O staining revealed that Sema3A overexpression reduced the adipogenesis of ASCs (Figures 2(e) and 2(f)). The Sema3A induced osteogenesis shift was further confirmed by qPCR analysis of the osteogenic and adipogenic marker genes.

As expected, osteogenic markers, including ALP, Runx2, and Col1a1, were significantly higher in Sema3A overexpressing ASCs, after both 5-day and 10-day osteogenic inductions (Figures 2(g)–2(i)). In contrast, the adipogenic marker genes, including FABP4, PPARG, and C/EBPA, were significantly suppressed by Sema3A expression after both 3-day and 5-day adipogenic inductions (Figures 2(j)–2(l)). Consistent with the osteogenesis promoting role of Sema3A, knockdown of Sema3A in BMSCs by siRNA significantly decreased the osteogenic capacity of BMSCs, as revealed by the Alizarin Red staining (Supplementary  Figures  3A–C).

### 3.3. PLGA Scaffold Encapsulated with Sema3A Modified ASCs Promotes Bone Regeneration In Vivo

**Figure 3:**
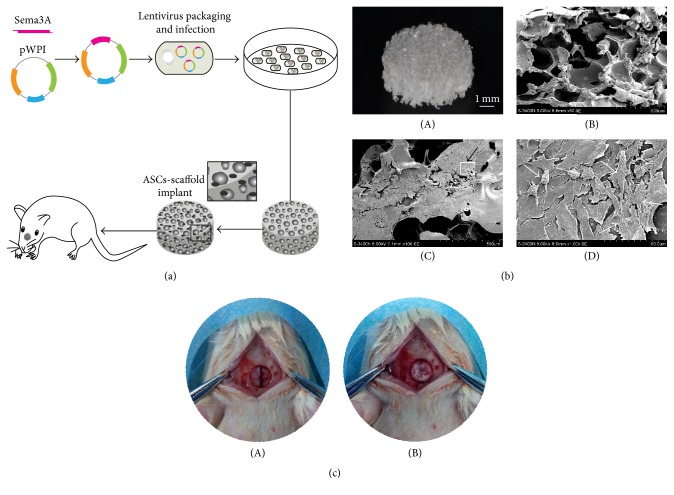
The fabrication of PLGA scaffolds engrafted with ASCs. (a) Schematic diagram of experiment procedure. (b) The morphology of PLGA scaffold-ASCs implants: (A) gross observation, (B) the lateral view of the scaffold without cells observed by SEM, (C) the surface view of the scaffold-ASCs implants under SEM, and (D) magnification to the inset in Figure 3(b)(C) and the cells were indicated by white arrow. (c) The critical size bone defect before (A) and after the transplantation of scaffold- ASCs implants (B).

We next explored whether Sema3A modified ASCs could promote bone regeneration in vivo in the calvarial bone defect model, with the detailed procedure summarized in Figure 3(a). PLGA scaffold was fabricated in a round mold, with the diameter of 5 mm and thickness of 1 mm, and the porous structure of the scaffold was achieved by soaking the sodium particles into the PLGA solution. SEM analysis confirmed the porous structure (Figure 3(b)). After culture of the ASCs on the scaffold for about 24 hours, abundant ASCs attached and spread out onto the porous surface of the scaffold, as observed under SEM (Figure 3(b)). Then, the scaffolds were transplanted into the bone defect region, which fitted well with the critical size bone defect (Figure 3(c)).

**Figure 4:**
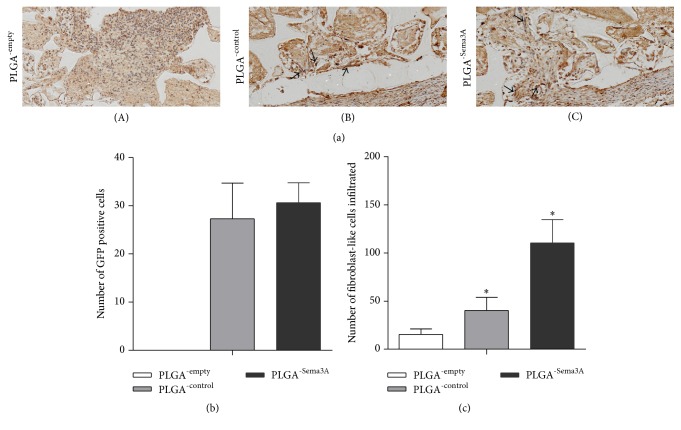
Sema3A infected ASCs decrease the immune cell infiltration while increasing fibroblast-like cells migrated to the implants. (a) The scaffolds were harvested 5 days after transplantation and immune-stained with collagen. (A) No GFP^+^ fibroblast-like cells were found in the empty implants and multiple inflammatory cells were shown, (B) a small fraction of fibroblast-like cells were found in implants seeded with ASC^-control^, and (C) Multiple fibroblast-like cells were found migrated and migrating towards the scaffold in implants seeded with ASC^-Sema3A^. (b) Quantification data of survival GFP^+^ ASCs in the scaffold. (c) Quantification data of migrated fibroblast-like cells. Mean ± SD, *n* = 3, and ^*∗*^ *p* < 0.05.

Five days after transplantation, the scaffolds were harvested for analyzing the cell survival and infiltration. As seen in the histology and collagen staining of the scaffold sections, there were abundant immune cells infiltrated in the PLGA scaffolds without ASCs. In contrast, there are few immune cells infiltrated in the PLGA scaffolds encapsulated with control or Sema3A modified ASCs (Figure 4(a)). Additionally, Sema3A modified ASCs recruited abundant collagen positive cells towards the scaffold (Figures 4(a) and 4(c)). Notably, Sema3A did not alter the survival of encapsulated ASCs in the scaffold, as no significant differences in the number of fluorescent positive cells were observed between the implants loaded with ASCs^-Sema3A^ or ASCs^-control^ (Figure 4(b)).

**Figure 5:**
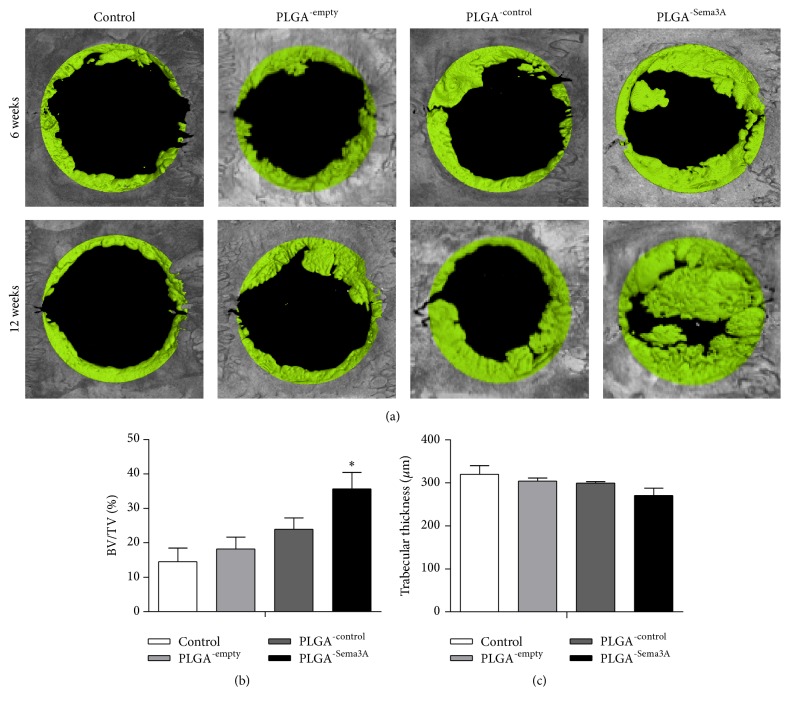
ASC^-Sema3A^ engrafted PLGA scaffold increases bone repair in the calvarial defect model. (a) Representative images showing bone defect healing after 4 or 16 weeks of treatments. Control indicates that defects received nothing; PLGA^-empty^, PLGA^-control^, and PLGA^-Sema3A^ indicate that defects implanted with ASC, ASC^-control^, and ASC^-Sema3A^, respectively. BV/TV (b) and trabecular thickness (c) of the regenerated bone 16 weeks after transplantation. Mean ± SD, *n* = 5, and ^*∗*^ *p* < 0.05. BV, bone volume; TV, total volume.

Furthermore, we analyzed the bone repair efficacy in vivo at both 4 weeks and 12 weeks after transplantation by histology and microCT. MicroCT reconstruction images indicated that the PLGA scaffolds encapsulated with ASCs^-Sema3A^ induced the largest new bone formation at both 4 weeks and 12 weeks, with minimal defects left at 12 weeks after transplantation (Figure 5(a)). Different from the Sema3A group, PLGA only and PLGA with control ASCs only had marginal bone repair promoting effects (Figure 5(a)). Quantitative analysis further revealed that PLGA scaffolds encapsulated with ASCs^-Sema3A^ increased the bone volume fraction but may not by changing trabecular thickness (Figures 5(b) and 5(c)).

**Figure 6:**
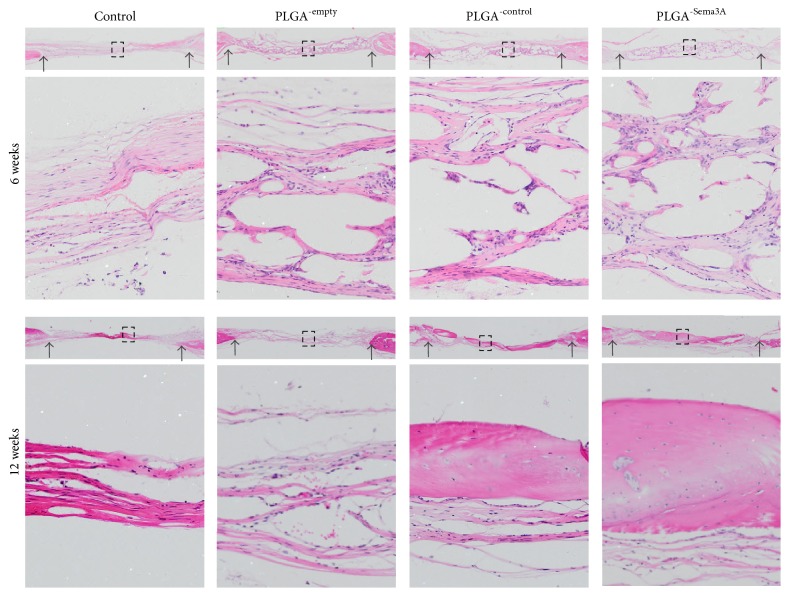
ASC^-Sema3A^ engrafted PLGA scaffold increases bone repair in the calvarial defect model. Bone regeneration of calvarial defects evaluated by HE staining. Control indicates that defects received nothing; PLGA^-empty^, PLGA^-control^, and PLGA^-Sema3A^ indicate that defects implanted with ASC, ASC^-control^, and ASC^-Sema3A^, respectively. Data presented are representative HE staining images from 5 different experiments. Black arrows point to the defect margins, and the lower panels are the magnifications of the insets in each group.

Consistent with the microCT findings, histology analysis revealed that PLGA scaffold degraded significantly at 4 weeks after transplantation, and meanwhile new bone formation began (Figure 6). Although no significant differences were found in the matrix deposited at 4 weeks after transplantation, there was a trend that there were more cell components and matrix in the PLGA scaffolds encapsulated with ASCs^-Sema3A^. Twelve weeks after transplantation, the ASCs^-Sema3A^ scaffold group showed the most abundant regenerated bone, which almost filled up the cranium defect. In contrast, the newly formed bones were much less in other groups, although ASCs^-control^ scaffold group also had robust bone repair when compared with the control and PLGA only group (Figure 6).

### 3.4. Sema3A Reprograms the ASCs towards BMSCs at Least Partially via Wnt Activation

**Figure 7:**
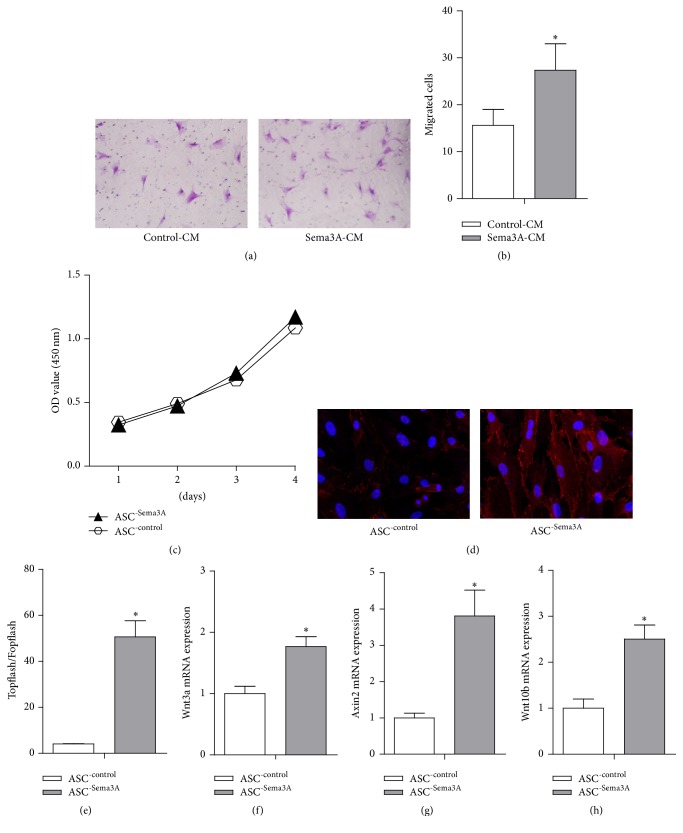
Sema3A significantly activates Wnt/*β*-catenin pathway in ASCs. (a) Conditioned medium from the Sema3A infected cells increased the migrated cells number. (b) Quantitative analysis of migrated cells in Figure 7(a). (c) No significant differences were found in the proliferation rate between ASC^-control^ and ASC^-Sema3A^. (d) Immunofluorescence staining of *β*-catenin in control and Sema3A overexpressed ASCs, and Sema3A induced nuclear translocation of *β*-catenin. (e) Sema3A overexpression significantly increased the Topflash/Fopflash ratio, indicating activation of Wnt pathway. (f, g, and h) The mRNA expression analysis of the Wnt pathway related genes and downstream transcriptional targets of *β*-catenin in control and Sema3A overexpressed ASCs. Mean ± SD, *n* = 3, and ^*∗*^ *p* < 0.05. CM, conditioned medium.

From the above PLGA scaffold mediated bone regeneration data, we could see that Sema3A promotes bone regeneration in vivo via shifting ASCs towards BMSCs. Besides cell fate shift, Sema3A seems inducing cell recruitment while not changing the ASCs numbers in vivo. To further explore the underlying mechanisms, we then tested the effects of Sema3A on proliferation and migration. Consistent with in vivo data, no significant difference on proliferation was observed upon Sema3A overexpression (Figure 7(a)). Furthermore, the conditioned medium from Sema3A infected ASCs induced a significant increase of mesenchymal stem cell migration (Figures 7(b) and 7(c)). Taken together, we could draw the conclusions that Sema3A promotes bone regeneration in vivo via simultaneously shifting ASCs towards BMSCs and inducing robust mesenchymal stem cell recruitment.

Previously, Wnt pathway was found to be essential in tipping the balance towards osteogenesis in mesenchymal stem cells [33]. We thus tested whether Wnt pathway was involved in the function of Sema3A. Topflash/Fopflash plasmids, a reporter system for Wnt activity, were included. As shown from the TOP/FOP ratio, Sema3A significantly increased the Wnt activity (Figure 7(d)). Consistent with the reporter assay, nuclei *β*-catenin staining was significantly increased in the Sema3A infected ASCs (Figure 7(e)). In addition, a few upstream and downstream Wnt pathway genes, like Wnt3a, Wnt10a, and Axin2, were found to be increased in Sema3A infected cells (Figures 7(f)–7(h)). All of these data sets suggest that Sema3A activates Wnt pathway through coordinately regulating multiple Wnt pathway genes.

## 4. Discussion

Our study here for the first time indicates that the implants combining the Sema3A modified ASCs with osteoinductive PLGA scaffolds successfully promote bone defect repair in a critical size cranium defect model, as revealed by both micro-CT and histological evidence. Mechanistically, Sema3A acts as a potent osteogenesis inducer via reprogramming ASCs towards osteogenesis in a Wnt dependent manner.

Bone defect repair capacity is affected by various factors including the age, metabolic status of the subjects, and the site, size, and shape of the injury [34–36]. Critical size bone defect could not be repaired without medical intervention. Current therapeutic approaches include autogenous or allogeneic bone graft and bone substitutes [2, 3]. In the bone substitutes field, tissue engineering bone has become the focus, and PLGA is an excellent osteoinductive scaffold candidate with porous structure, excellent compatibility property, and some plasticity. Accordingly, our in vivo and vitro studies here further confirmed the applicability of PLGA scaffold in bone repair and regeneration.

Besides the scaffold, the seeding cell is another essential component of the tissue engineered bone substitute. The origin and identity of the seeded cells directly influence the output of tissue engineered bone [37]. Seed cell usually refers to stem cells in tissue engineering, which contains various types, among which BMSCs and ASCs are the common ones2. Both BMSCs and ASCs are thought mainly of pericyte origin [38, 39] and consist of an heterogeneous cell populations without specific cell markers [40–42]. These two cell types also display distinct biological features. Previous researches have shown that the differentiation tendency of mesenchymal stem cell is related to its tissue origin, with BMSCs tending to differentiate into osteoblasts, while ASCs are sensitive to adipogenic differentiation [11, 13]. BMSCs work efficiently in bone repair as observed in animal experiments and clinical studies [43]; however, the limited resources restrict its clinical application. Different from BMSCs, ASCs are readily available and closely related to BMSCs. Identification of the molecules responsible for the differences would certainly shed light on fine-tuning the cell identity towards the destination beneficial for bone regeneration. We here screened the semaphorin family members in BMSCs and ASCs. Among the semaphorins 3A, 3B, 3C, 3D, 3E, 3F, 4A, 4B, 4C, 4D, 4F, 4G, 5A, 5B, 6A, 6B, 6C, 6D, and 7A, Sema3A was identified as the key differential expression molecule. Consistent with previous concept that Sema3A promotes osteogenesis [24], we also found that restoration of Sema3A in ASCs promotes ASC osteogenic differentiation. Moreover, we found that expression of Sema3A in ASCs from 12-month old rats was further decreased, when compared with that in ASCs from 4-week old rats. All of these data sets confirm that Sema3A acts as a key determining factor for the different osteogenic capacity between BMSCs and ASCs. Accordingly, we transfected ASCs with Sema3A and explored its capacity in bone defect repair. In vitro analysis revealed that ASCs^-Sema3A^ increased the osteogenesis, migration, concomitant with decreased adipogenic potential. ASCs^-Sema3A^ grafted PLGA scaffold exhibits distinctly enhanced bone matrix secretion in the cranial bone defect model. These functional changes initiated by Sema3A transfection indicate that Sema3A could induce a BMSCs phenotype in ASCs.

To further clarify the detailed molecular mechanism of how Sema3A reprograms ASCs to BMSCs like phenotype, we here focused on the role of Wnt/*β*-catenin signaling pathway which is closely relevant to both osteogenic differentiation and adipogenic differentiation [33, 44]. Our data revealed that Wnt/*β*-catenin activation was at least partially involved in the function of Sema3A. The findings here are consistent with the accumulating evidence revealing that Wnt is essential for promoting osteogenesis and inhibiting adipogenesis [33, 44]. However, we cannot exclude other possible signal molecules involved. In fact, Sema3A itself is an endocrine factor and could function as a chemotaxis inducer in different systems [44]. It is thus reasonable to hypothesize that increasing cell recruitment of endogenous MSCs might be another function of Sema3A modified ASCs in our system. Systemic analysis of the gene expression of ASCs, Sema3A modified ASCs, and BMSCs would further solidify our conclusion. In addition, encapsulation of Sema3A factor in the PLGA scaffold in cranial bone defect model would further pave the way for Sema3A application in bone repair.

## 5. Conclusion

Our study here has identified that high expression of Sema3A in BMSCs is essential for its potent osteogenic capacity, which functions as a determinant of the BMSC phenotype. Transferring Sema3A into ASCs at least partially reprograms the cells towards BMSCs and thus increases the osteogenic capacity via Wnt activation and possibly other mechanisms. Encapsulation of Sema3A modified ASCs in the PLGA scaffold significantly increased the bone defect repairing efficacy, which provides a promising strategy to overcome the limit resource of BMSCs in bone regeneration and repair.

## Supplementary Material

The supplementary data include three figures and one table. Supplementary Figure 1 refers to the detailed expression of semaphorin family members (Semaphorin 3A, 3B, 3C, 3D, 3E, 3F, 4A, 4B, 4C, 4D, 4F, 4G, 5A, 5B, 6A, 6B, 6C, 6D, and 7A) in ASCs and BMSCs and in BMSCs from different age groups. Supplementary Figure 2 depicts the overexpression efficiency of Sema3A as confirmed by flow cytometry, Q-PCR and Western blot. Supplementary Figure 3 refers to the role of Sema3A in BMSC osteogenesis via knockdown of Sema3A. Supplementary Table 1 covers all the primers used in the study.
