# Supplementary material for: Semaphorin 3A Shifts Adipose Mesenchymal Stem Cells towards Osteogenic Phenotype and Promotes Bone Regeneration In Vivo
Source: Stem Cells Int. 2016 Sep 19;2016:2545214. doi: 10.1155/2016/2545214 (PMC5046026; doi:10.1155/2016/2545214)
Supplement: Supplementary file 1 — The supplementary data include three figures and one table. Supplementary Figure 1 refers to the detailed expression of semaphorin family members (Semaphorin 3A, 3B, 3C, 3D, 3E, 3F, 4A, 4B, 4C, 4D, 4F, 4G, 5A, 5B, 6A, 6B, 6C, 6D, and 7A) in ASCs and BMSCs and in BMSCs from different age groups. Supplementary Figure 2 depicts the overexpression efficiency of Sema3A as confirmed by flow cytometry, Q-PCR and Western blot. Supplementary Figure 3 refers to the role of Sema3A in BMSC osteogenesis via knockdown of Sema3A. Supplementary Table 1 covers all the primers used in the study. [file 2545214.f1.docx]

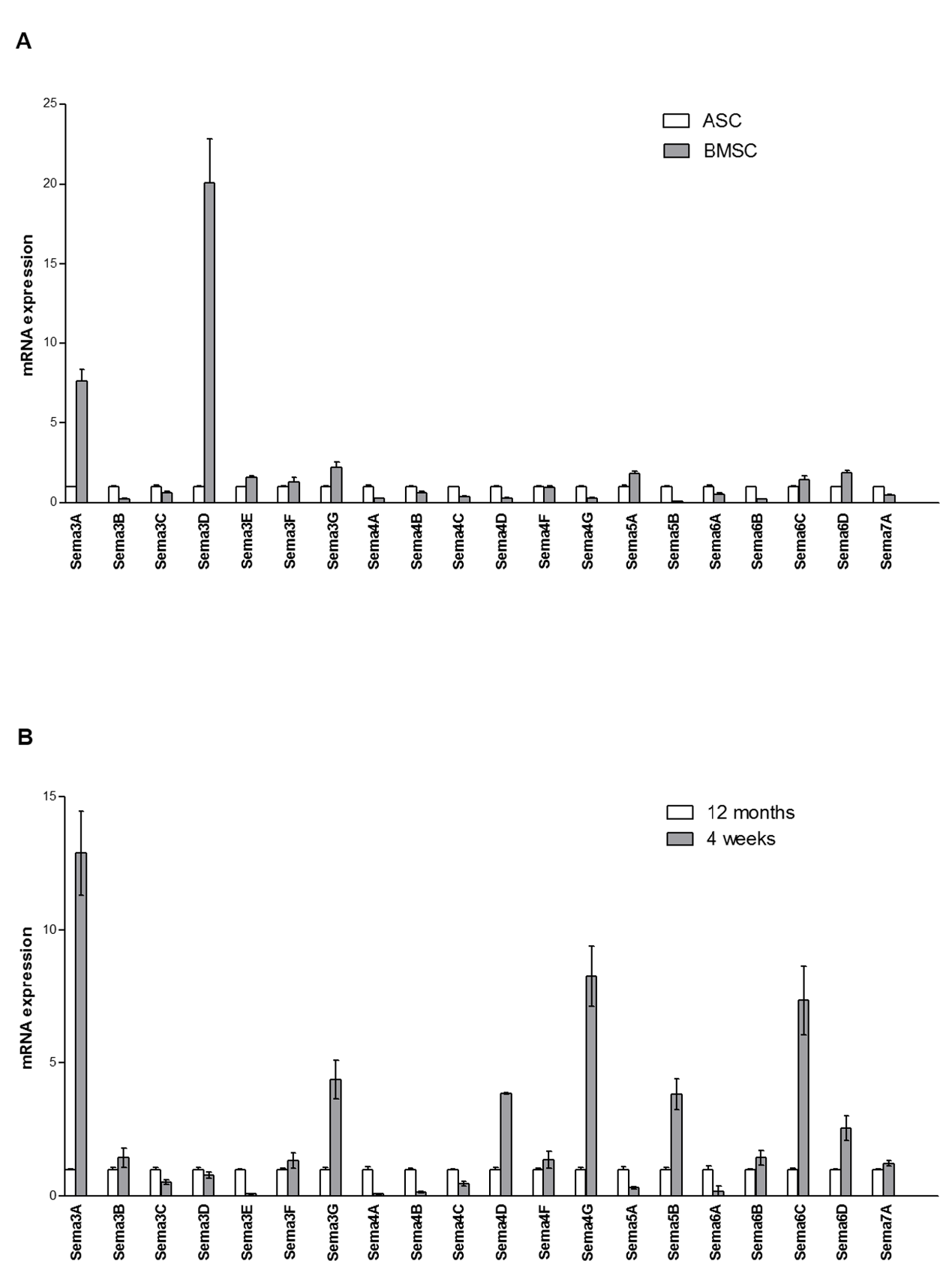


**Supplementary Figure 1.** The mRNA expression of Semaphorin family members between ASCs and BMSCs derived from the same rats (A), and in BMSCs from either 4 weeks or 12 months old rats (B). mean±SD, n=3, * p<0.05.


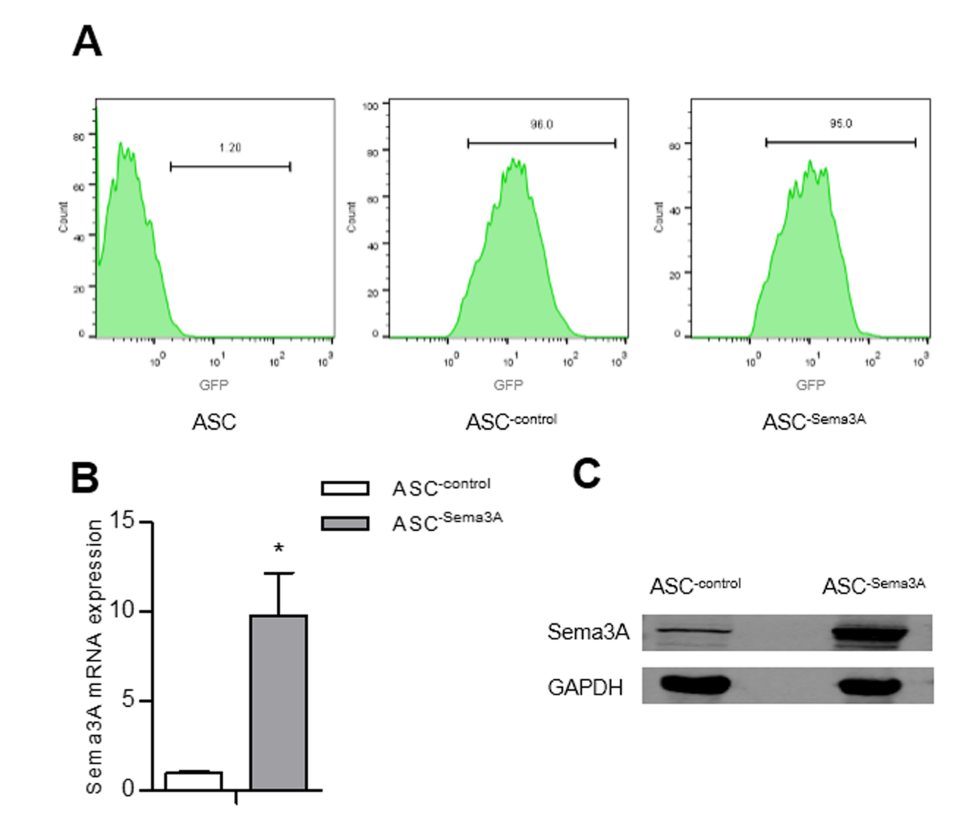


**Supplementary Figure 2. Validation of Sema3A overexpression in ASCs.**

1. Flow cytometry showed that most of the ASCs are GFP^+^ after control or Sema3A expressing lentivirus infection. (B) qPCR analysis confirmed significant increase of Sema3A in ASC^-Sema3A^. mean±SD, n=3, * p<0.05. (C) Western blot analysis confirmed significant increase of Sema3A in ASC^-Sema3A^. Representative data of three different experiments.


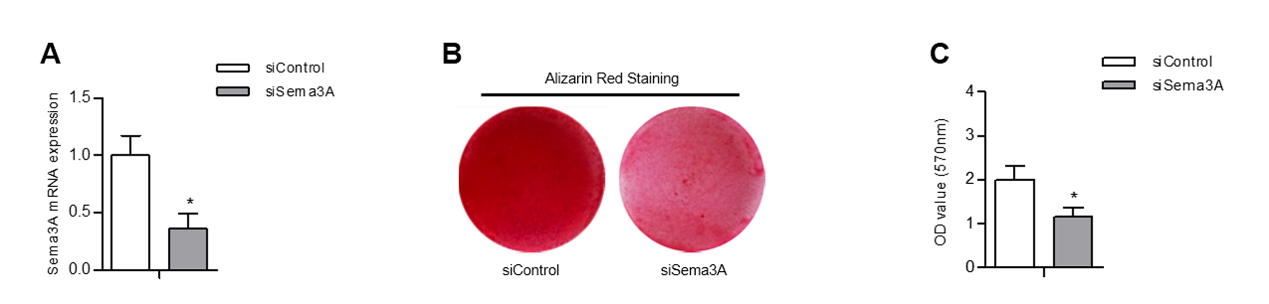


**Supplementary Figure 3. Knockdown of Sema3A inhibits osteogenic differentiation in BMSCs.**

(A) Sema3A expression was significantly reduced in BMSCs by siSemaA as detected by qPCR. (B) Significantly less mineralization nodules were found in BMSCs treated with siSema3A than that in the control BMSCs after 21 days of osteogenic induction. (C) Quantification data of ECM mineralization in Supplementary Figure 3B.

**Supplementary Table 1.** Primers for real-time quantitative polymerase chain reaction

| ALP | ACAACCTGACTGACCCTTCCC | CAATCCTGCCTCCTTCCACTA |
| --- | --- | --- |
| COL1a1 | AAGGCAATGCTGAATCGTCC | TGGTTAGGCTCCTTCAATAGTCC |
| RUNX2 | GAACAGTAGCGGGAGCAT | CCCACATAGGAGGAGAAAA |
| FABP4 | ACACCGAGATTTCCTTCAAT | CCACCATCCAGGGTTATG |
| PPARG | ACAATGCCATCAGGTTTGG | GCGGGAAGGACTTTATGTATG |
| C/EBPα | TTCCTGGCTGACTTATTCC | CTCCCGAGGCTCTTGTT |
| Wnt3a | TTAATGACAGGGCACTAACAA | CTGGCATCGGCAAACTC |
| Wnt10b | CGCTGCCACTGTCGTTTC | CATCCTAGTCTATTCGGGTGTTTA |
| Axin2 | AAACAGACGACGAAGCACG | GGCAGACTCCAACGGGTAG |
| Sema 3A | TATGAGTGATGTAAGAAGGGTG | CTGGTCGTGGATAAGGGA |
| Sema 3B | CGAGGATGGCAAGGGAAAG | CGGACTCACGGAAGAAGAAATAG |
| Sema 3C | AAGTCTCCGCAGGCATCTAT | GAATCTTGAACAGAGCGGAT |
| Sema 3D | CTCGGAACAGACATTGGAAC | GTTGGGTGCTTGAATACCTG |
| Sema 3E | GCGTCAGTGATGGCTACAGA | CGTAGCACAGGTCAGAAGGT |
| Sema 3F | TGTCACTGGCTTCATCCTCTG | GTTGAAGAAGTGGGCAGTACC |
| Sema 3G | GGTGTGTGTCCCAGCAAGAT | CACCACGATCTGGCACAGTT |
| Sema 4A | GTGCCCAGAGTCAAGTACCA | CGAGCCCCCACATACAGAGT |
| Sema 4B | GGCTCTGGTGGTTGATGGTG | CCCAGGCTCTCAGGAACATA |
| Sema 4C | AGACAGGCATCCAGGACTTC | CACTCGGTCTGGTTGCTCTT |
| Sema 4D | AAGTGGGTACGCTACAATGG | CTGTTGTCTATTGGCGTCAC |
| Sema 4F | TCTACACCGCCACTGTGAAG | GCGACAAAGGCTGGAGCATT |
| Sema 4G | TGCCCTGTTCTCTCTCAGTG | AGCCGCTGTAGAAATCGCAC |
| Sema 5A | AGTGTGATGAAGCCACGAAG | TAAGGAGCGGATGGTACAGA |
| Semp 5B | GCCTTCAACCTCAGTGCCAT | TCCGTGAGGTTCTCGTTAGG |
| Sema 6A | CTCCACTGCCTCGGTATCCA | GTGAGTCCGACCTGCTGTAT |
| Sema 6B | TGCCCTTCCTGACGAGATTC | ACTGTGCCAACCTCAGAGCC |
| Sema 6C | GTTCAAGGAGCAGAGGAGTC | AGAGCAGGACATCGTCAGGC |
| Sema 6D | GGACTAAAGAGGACACCATC | TAAAGCCACACAGGAATAGC |
| Sema 7A | TAATGGACTGCTGGTCTGTG | CATCCCCTTCAAACAGAACC |
| GAPDH | GGTGCTGAGTATGTCGTGGAG | GCGGAGATGATGACCCTTTT |
| β-actin | AATCGTGCGTGACATTAAAGAG | CATTGCCGATAGTGATGACCT |

**Abbreviations:** AlP, alkaline phosphatase; COL1a1, collagen, type I, alpha 1; RUNX2, runt-related transcription factor 2; FABP4, fatty acid binding protein 4; PPARG, peroxisome proliferator-activated receptor gamma; C/EBPA, CCAAT/enhancer binding protein alpha; Wnt3a, wnt family member 3A; Wnt10b, wnt family member 10B; Sema, semaphorin; GAPDH, glyceraldehyde-3-phosphate dehydrogenase;β-actin, actin beta.
